# Analysis of Soft Tissue N-Glycome Profiles in Oral Squamous Cell Carcinoma, a Pilot Study

**DOI:** 10.3390/ijms27020740

**Published:** 2026-01-11

**Authors:** Eniko Gebri, Kinga Hogyor, Adrienne Szabo, Andras Guttman

**Affiliations:** 1 Department of Oral Medicine, Faculty of Dentistry, University of Debrecen, H-4032 Debrecen, Hungary; gebri.eniko@dental.unideb.hu; 2 Translational Glycomics Group, Research Institute of Biomolecular and Chemical Engineering, University of Pannonia, H-8200 Veszprem, Hungary; hogyorkinga@gmail.com; 3 Department of Oral and Maxillofacial Surgery, Faculty of Dentistry, University of Debrecen, H-4032 Debrecen, Hungary; szabo.adrienn@dental.unideb.hu; 4 Horváth Csaba Memorial Laboratory of Bioseparation Sciences, Research Center for Molecular Medicine, Faculty of Medicine, University of Debrecen, H-4032 Debrecen, Hungary

**Keywords:** N-glycome, biomarkers, oral squamous cell carcinoma, capillary electrophoresis, glycogens

## Abstract

Oral squamous cell carcinoma (OSCC) is an aggressive disease with a glycoproteomically unmapped progression and a low five-year survival rate. Thus, the aim of this pilot study was to explore the N-glycosylation pattern differences in malignant, adjacent mucosal and healthy tissues in the context of OSCC. Oral mucosal soft tissue samples was obtained by incisional biopsy from five patients with OSCC, both from the malignant and the opposite healthy gingival sides, and from seven age-sex-matched healthy controls. The collected tissues were homogenized, followed by N-glycan profiling of the endoglycosidase-released and fluorophore-labeled carbohydrates using capillary electrophoresis with ultra-sensitive laser-induced fluorescent detection (CE-LIF). Six out of the twenty-two identified N-glycan structures, including glycogens, showed significant (*p* < 0.05) differences between the malignant tissue samples of the OSCC patients and the healthy controls. Comparing the healthy and the positive control oral mucosal samples, differences in four N-glycan structures were revealed, while only one alteration was observed between the N-glycan profiles of the malignant tumor and positive control samples. However, the results are presented descriptively, reflecting the limited sample size of the pilot study, it shows the potential of high-resolution CE-LIF-based glyocoanalytical protocol to be highly efficient and sensitive for glycobiomarker-based molecular diagnostics of oral malignant lesions.

## 1. Introduction

In the past 50 years, the mortality rates of oral cancers have increased almost six-fold and disability-adjusted life-years of oral cancer patients have also shown a slight elevation trend [1]. While oral cancer is only the sixteenth most common malignancy worldwide, it has the highest incidence and mortality rate in Asia and the sixth for both sexes in Hungary [2,3]. More than 90% of oral cancer is squamous cell carcinoma (OSCC) and as the number proliferates, more than 377,000 new cases are diagnosed and consequently, 170,000 deaths are recorded worldwide annually. More importantly, OSCC has a relatively low (40–70%) five-year overall survival rate and interestingly has a higher prevalence in males than females. The tumor occurs mainly in the sixth and seventh decades of life, although the number of younger patients under 45 years is also increasing, being mostly associated with human papilloma virus (HPV) infection [2,3]. Squamous cell carcinoma not only affects the oral cavity but also the oropharyngeal region (OP-SCC), which shows a dramatic increase in its incidence in the US, again, due to HPV infection [4]. While the oral cavity consists of the labial mucosa, floor of the mouth, anterior two-thirds of the tongue, alveolar ridge and gingiva, buccal mucosa, hard palate, and retromolar trigone; the oropharynx includes the soft palate, palatine tonsils, palatoglossal folds, valleculae, posterior pharyngeal wall and the base of the tongue. The tumors at these two sites have different etiopathogenesis, treatment and prognosis [4]. The tumor on the tongue is the most common subsite and predicts higher mortality than other sites. Perineural spread, bone involvement and lymph node metastasis suggest poor prognostic value [5]. Besides the most commonly known risk factors, such as alcohol consumption, tobacco, poor oral hygiene and HPV infection, long-term immunosuppressant therapies may also increase the risk and change the therapeutic response of secondary malignancies [2,6]. Many factors determine the choice of the optimal treatment alternative, such as primary tumor site, stage, resectability, and patient factors. Surgical intervention and radiotherapy are the major treatment options combined with chemotherapy based on appropriate indication [7]. With new developments in oncotherapy, monoclonal antibodies have also found their place in the treatment of head and neck cancer. Cetuximab, an epidermal growth factor receptor inhibitor monoclonal antibody has been approved to apply in combination with radiation in HPV-negative OSCC, while pembrolizumab is an immune checkpoint inhibitor, used as a primary treatment option for unresectable disease and recurrent or metastatic OSCC [8]. A deeper understanding of the molecular pathways of OSCC can help in developing less toxic, more targeted therapeutic alternatives and identifying predictive markers that can be used in clinical practice.

Oral cancers are often detected at advanced stages only [9]. Therefore, it would be particularly important to discover early, potentially non-invasive biomarkers for oral cancers. However, many omics (genomics, transcriptomics, proteomics, metabolomics) focus on identifying sufficiently sensitive, specific and reliable markers, suitable for monitoring therapeutic response or early detection of tumor recurrence in addition to early diagnosis [10,11,12,13], the number of validated clinically useful biomarkers is negligible for the time being [14]. Saliva analysis can also be an excellent approach for this, as it also reflects detectable changes in tissues [15,16].

Glycosylation is one of the most common post-translational modifications, which has a pivotal role in several important biological processes such as cell-cell adhesion, mediating and regulating cell-matrix interactions, affecting protein stability and solubility, or information transmission at the intra- and extracellular level [17]. Aberrant glycosylation, on the other hand, is often observed in inflammation, tumorigenesis and metastasis formation [18], therefore, holds biomarker potential. Hypersialylation, e.g., a special modification, which is often related to tumorigenesis, progression and prognosis [19]. Increased free serum sialic acid can be observed in several solid tumor cases, such as oral cancer, while the alteration of N-glycan associated sialylated structures may reflect the effectiveness of therapeutic response to various oncological treatments. Numerous studies examined the changes of the total sialic acid level, sialyltransferase activity, sialidase activity, total sialic acid to total protein ratio in serum [20,21,22], saliva [23] or both [24,25,26] and soft tissue [12] in patients with oral cancer, and oral premalignant disorders compared to healthy controls. These studies consistently confirmed that the variables examined showed significantly elevated levels in cancer patients and these changes showed positive correlation with tumor stage, grade, progression and therapeutic response. Besides altered terminal sialylation, fucosylation has also been reportedly associated with tumor formation [18]. Significantly increased fucose level and mRNA expression of a specific fucosyltransferase enzyme (FUT6) were reported in patients with oral cancer compared to controls [27]. In addition, these authors observed increased fucose level and more pronounced FUT6 mRNA expression in poorly differentiated and advanced stage tumors than in well-differentiated and early stage tumors. Meanwhile, others highlight the biomarker role of fucose in the grading of oral cancer [28], and revealed that increased fucosylation has a pivotal role in invasive and metastatic properties of head and neck cancer stem cells [29]. Although extensive glycomic studies are underway to understand the etiology [30], development and progression of oral cancers and to find potential biomarkers [31], the number of deeper N-glycan analyses is relatively low [32].

Recent developments in separation science have transformed the landscape of glycomics, enabling high-resolution structural characterization of N-glycans even from small tissue samples with high sensitivity [33]. Hydrophilic interaction chromatography (HILIC), porous graphitized carbon liquid chromatography (PGC-LC), mass spectrometry (MS) and capillary electrophoresis with laser-induced fluorescence detection (CE-LIF) have all contributed to a quantitative and structurally detailed understanding of glycosylation in biological specimens [34]. In particular, CE-LIF in combination with exoglycosidase enzyme digestions and computational tools for structural annotation provided an opportunity to map comprehensive glycan signatures associated with malignant transformation [35,36]. Most importantly, these profiling approaches can detect subtle changes in branching patterns, terminal modifications, and glycan microheterogeneity—features that are often critical for distinguishing between normal and malignant conditions.

In this paper, we introduce a novel characterization approach of N-glycan alterations intrinsic to OSCC and evaluate their predictive potential in oral oncology. It should also be noted that this is the first report using capillary electrophoresis-based glycan analysis to reveal the biomarker potential of cellular glycogen concentration changes in oral cancer.

## 2. Results

**Figure 1:**
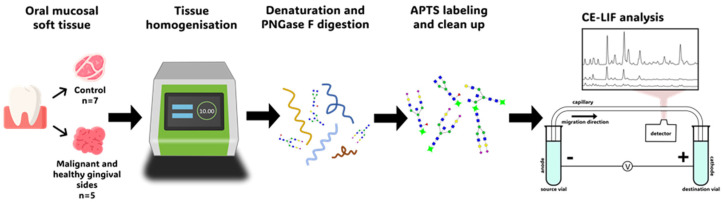
The glycoanalytical workflow includes soft tissue sample collection (step 1), automated tissue homogenization (step 2), N-glycan release (step 3), fluorophore labeling (step 4) and capillary electrophoresis separation with laser-induced fluorescence detection (step 5).

The goal of our exploratory study was to evaluate a novel capillary electrophoresis-based workflow to identify novel glycobiomarker candidates that support high-precision prognosis and provide efficient therapeutic alternatives for oral cancers. The developed workflow, shown in Figure 1, comprised automated homogenization of the collected soft tissue samples, N-glycan release, and fluorophore labeling, followed by CE-LIF-mediated glycoprofiling. 

**Figure 2:**
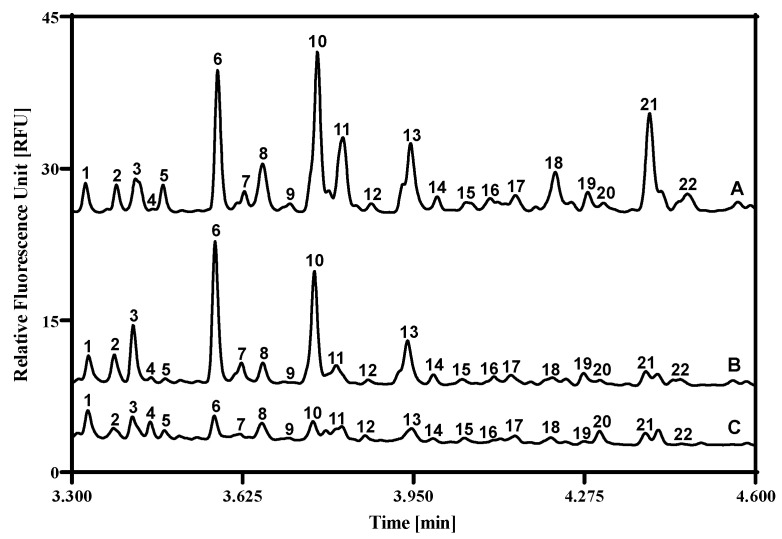
Comparative capillary electrophoresis analysis of the representative carbohydrate profiles (including N-linked glycans and glycogens) of the healthy control (trace A, ST-1, C5), as well as the healthy (trace B, ST-1, OSCC-C2) and malignant (trace C, ST-1, OSCC-T2) gingival sides of an oral squamous cell carcinoma (OSCC) patient. The glycan structures corresponding to the numbered peaks are listed in Table 1. Conditions: Separation capillary: 20 cm effective length BFS (30 cm total, 50 μm i.d./365 μm o.d.) filled with HR-NCHO gel buffer; Applied electric potential: 30 kV in reversed polarity mode; Separation temperature: 25 °C; Injection: water plug: 5 psi/5 s followed by the sample: 2 kV/2 s.

Using the above introduced workflow, first, the asparagine-linked carbohydrate profile of the healthy control samples were compared to the tissues obtained from the healthy and malignant gingival sides of the oral squamous cell carcinoma (OSCC) patient (Supplementary Table S1). Figure 2 shows the resulting electropherograms of a representative example. Twenty-two peaks were identified with >1% relative peak area in any of the three traces. Comparing the healthy control to the healthy gingival side, one can observe an increase (↑) in the area of peak 3↑, and a decrease (↓) of peaks 5↓, 8↓, 11↓, 18↓, 21↓, and 22↓. On the other hand, comparison of the healthy control trace to the malignant side sample revealed increased areas of peaks 4↑ and 20↑, but a decrease in peaks 2↓, 5↓, 6↓, 8↓, 10↓, 11↓, 13↓, 18↓, 21↓, and 22↓.

**Figure 3:**
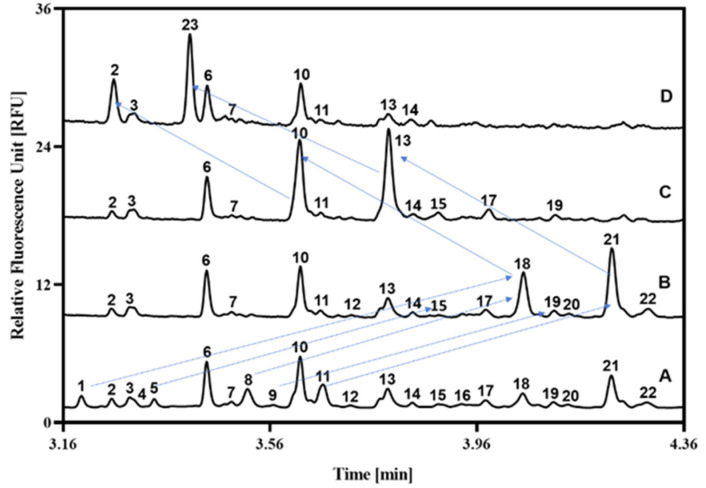
Exoglycosidase array-based carbohydrate sequencing for high precision structural identification. Traces: (A) control (ST-1, C5); (B) sialidase; (C) sialidase + galactosidase; (D) sialidase + galactosidase + hexosaminidase. The arrows highlight examples of digestion mediated consecutive structural changes. Peak assignment is depicted in Table 1. Conditions were the same as in Figure 2.

Comparing the healthy and malignant gingival sides of the OSCC patient samples, the following increasing and decreasing differences were observed: peaks 2↓, 3↓, 4↑, 6↓, 7↓, 10↓, 13↓, 19,↓ and 20↑. The structures corresponding to the numbered peaks were identified by using the GUcal ver 1.2 software (www.gucal.hu accessed on 23 November 2025), publicly available databases (Glycosmos.org), and exoglycosidase enzyme-mediated glycan sequencing. To assure precise annotation of the separated oligosaccharides, we used the latter for verification. Figure 3 depicts the sequencing traces after the administration of sialidase (trace B), sialidase and galactosidase (trace C), as well as sialidase, galactosidase, and hexosaminidase (trace D) enzymes. As one can observe, after the sialidase treatment (trace B), peaks 1, 5, 8, 9, and 11 shifted to 18, 15, 18, 19, and 21, respectively. After the combined treatment with sialidase and galactosidase (trace C), the two major peaks of 18 and 21 shifted to 10 and 13, respectively. The reaction mixture containing sialidase, galactosidase, and hexosaminidase (trace D) caused peak 10 to shift to peak 2, and peaks 13 to 23. Structural assignments of the numbered peaks are listed in Table 1.

**Figure 4:**
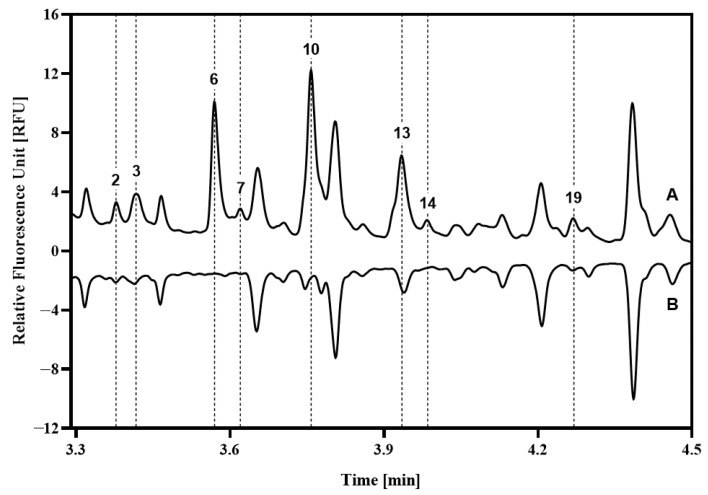
Effect of the amyloglucosidase treatment on the glycans derived from the sample of a representative healthy control patient. (A) control; (B) digested sample. Peak assignment is depicted in Table 1. The dotted lines guide the eye for the depletion of the glycogen content of the peaks. Conditions were the same as in Figure 2.

Interestingly, peaks 2, 3, 6, 7, 10, 13, 14, and 19 were not or were only partially affected by these three digesting enzymes. With the consideration of the possibility of the presence of glycogen fragments in the cells of the collected soft tissue samples, the control sample was treated with amyloglucosidase, as shown in Figure 4. The butterfly diagram clearly reveals the disappearance of the majority of these peaks, except for peak 13, in which instance approximately 1/3rd of the peak remained intact.

**Figure 5:**
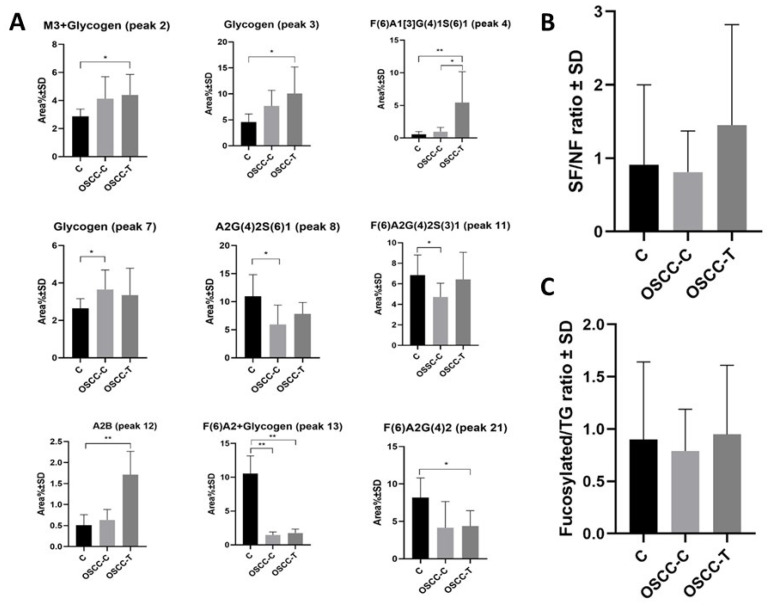
Descriptive visual summary of the (**A**) significantly changed carbohydrate structures (N-glycans and glycogens) in the three examined groups: OSCC-T: malignant tumor sides of the oral squamous cell carcinoma patients; OSCC-C: healthy gingival sides of the oral squamous cell carcinoma patients; C: healthy controls. (**B**) The sialoform to neutral (SF/NF) carbohydrate ratio (%) in the three examined groups; (**C**) The ratio of core-fucosylated structures to the total N-glycosylation in the three examined groups. (* *p* < 0.05, ** *p* < 0.01).

After careful statistical analysis of the data (Supplementary Table S2), besides two mostly glycogen related peaks (2 and 3), the N-glycan structures of F(6)A1[3]G(4)1S(6)1, A2B, F(6)A2, and F(6)A2G(4)2 out of the 22 identified oligosaccharides showed significant (*p* < 0.05) differences between the malignant tissue samples of OSCC patients and the healthy controls as depicted in Figure 5A. Comparing the healthy and the positive control oral mucosal samples, besides the glycogen peak (7), differences in the following N-glycan structures have been revealed: A2G(4)2S(6)1, F(6)A2G(4)2S(3)1 and F(6)A2, while only the F(6)A1[3]G(4)1S(6)1 glycan showed alteration between the N-glycan profiles of the malignant tumor and positive control samples.

We also evaluated the sialoform to neutral (SF/NF) (Figure 5B) and core-fucosylated (Figure 5C) to total N-glycan (TG) ratios (Supplementary Tables S3 and S4). In both instances, the malignant side of the OSCC patients (OSCC-T) samples showed an increasing tendency in comparison to the healthy gingival side (OSCC-C). Interestingly, the SF/NF and fucosylated/TG ratio values of the healthy control sample (C) were between the OSCC-C and OSCC-T levels. 

## 3. Discussion

This work aimed to provide the complete workflow for the exploratory characterization and future large-scale studies of the N-glycosylation patterns associated with OSCC using capillary electrophoresis-based carbohydrate profiling. The carbohydrate structures of interest (relative peak area > 1%) were validated by exoglycosidase array-mediated oligosaccharide sequencing. 

### 3.1. Mucosal Soft Tissue N-Glycosylation

The mostly biantennary type glycan structures identified in this study showed similarities to other reported works in serum, saliva and tissue samples [32,38,39]. Furthermore, the ratio of sialylated to neutral and fucosylated to total glycan structures showed an increasing trend in the tumor tissue, as is generally reported in malignant processes [27].

### 3.2. Glycogen Depletion

The rapid proliferation of tumors requires significant energy, so they quickly deplete cellular glycogen stores [40,41], as evidenced by the reduced glycogen levels in our data (Figure 4). Glycogen is a multi-branched oligosaccharide comprising α1-4 and α1-6 linked glucose molecules with an average chain length of approximately 8–12 units, serving as a form of energy storage in cells. Amyloglucosidase readily digests the building blocks of this oligosaccharide, but has higher specificity to the α1-4 linked ones; therefore, as a first approximation, we consider that the remaining peak portions after this digestion step contained a higher level of α1-6 linked glucose units. However, by all means, the major revelation of this phenomenon was that the different chain length glycogen oligomers were in the released N-linked glycan pool, labeled along with them by the fluorophore dye and subsequently detected during capillary electrophoresis analysis. Considering the above in view of the differential electrophoretic analysis shown in Figure 2, major changes were observed in the glycogen peaks (6, 10 and 13) in addition to the released N-linked sugars (3, 8, 18 and 21).

### 3.3. Study Limitations

Since this pilot study was performed on a limited number of samples, the results should be considered indicative rather than confirmatory. Continuation of this work, including a larger number of individually analyzed soft tissue samples amended with serum and saliva specimens, will be an important next step towards verifying the observed trends and elucidating their prospective biological significance.

### 3.4. Significance and Potential Benefits

Although our conclusions were drawn with caution, pointing out that future studies with larger cohorts and individual-level analyses will be required to validate and extend these observations, it is important to emphasize that this manuscript describes significant methodological achievements to help our peers in similar studies. The novel data generated by analyzing the soft tissue N-glycome in OSCC, even if somewhat limited, highlights the significance of the glycobiomarker discovery perspective of this new workflow. In addition, to the best of our knowledge, this is the first demonstration of the use of CE-LIF-based carbohydrate analysis to investigate cellular glycogen changes as potential biomarkers in malignant diseases, plausibly benefiting similar studies where glycogenic changes were not considered. In medical point of view, our study helps to map the OSCC glycome, thus understanding the role of N-glycans and glycogens in the etiopathogenesis of OSCC as well as to identify early, non-invasive biomarkers. 

## 4. Materials and Methods

### 4.1. Chemicals

Tetrahydrofuran (THF) and isopropyl alcohol (≥99.9%) were from VWR (Debrecen, Hungary). Acetic acid was from Molar Chemicals Kft. (Halasztelek, Hungary). The HPLC grade water, ammonium acetate solution (7.5 M), acetonitrile (≥99.5%), sodium-cyanoborohydride (1.0 M in THF) and trypsin (from bovine pancreas, ≥10,000 BAEE units/mg protein) were purchased from Sigma-Aldrich (St. Louis, MO, USA). The PNGase F (2.7 mg/mL), neuraminidase (0.2 mg/mL), galactosidase (0.34 mg/mL) and hexosaminidase (0.14 mg/mL) enzymes were provided by the Bio-Nanosystems Laboratory ( University of Pannonia, Veszprem, Hungary). The acidic (0.1 M HCl) and basic (0.1 M NaOH) wash solutions, the carbohydrate separation gel-buffer (HR-NCHO), 1-Aminopyrene-3,6,8-trisulfonate (APTS) and glycan capture beads were from Bio-Science Kft (Budapest, Hungary).

### 4.2. Tissue Sample Collection

Oral mucosal soft tissue samples were collected from five OSCC patients at the Department of Oral and Maxillofacial Surgery, Faculty of Dentistry , University of Debrecen (Debrecen, Hungary) by incisional biopsy from the malignant and the healthy sides of the gingiva, and their N-glycome profiles were compared with those of age- and sex-matched healthy controls. All OSCC patients have the same histopathological diagnosis of the tumor. The healthy control samples were derived from seven patients (collected at the Department of Dentoalveolar Surgery, Faculty of Dentistry, University of Debrecen (Debrecen, Hungary)), who were sex/age-matched (*p* = 0.0796) with the OSCC patients (Figure 1, step 1) having no acute or chronic diseases. The samplings were done during elective tooth extraction (with prosthetic or orthodontic indications) in case of healthy volunteers, while in the OSCC patients during the dental resolution prior to oncotherapy under local anesthesia. The samples were collected with the appropriate Ethical Permissions (Clinical Center, Regional and Institutional Research Ethics Committee, University of Debrecen, Hungary) and Informed Patient Consent forms (DE RKEB/IKEB: 6152-2022). The data of the OSCC patients and the controls are summarized in Table 2. 

### 4.3. Sample Preparation/Tissue Homogenization

**Figure 6:**
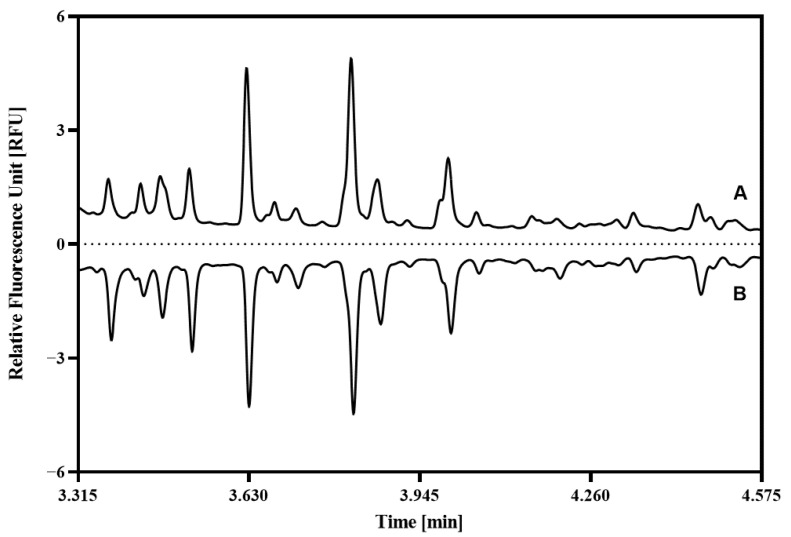
Comparison of the automated (A, BeatBox) and manual (B, Potter) tissue homogenization techniques.

Two milligrams of each tissue sample were homogenized by an automated tissue homogenizer (BeatBox, Tissue kit 96-wells, PreOmics, Munich, Germany). The homogenizer was run on the highest setting for 30 minutes (Figure 1, step 2). To verify the homogenization efficiency of the automated tissue homogenizer, blending was also performed using a manual Potter (Kontes Glass Co., 2 mL(witeg Labortechnik GmbH, Wertheim, Germany)). The weighed pieces of tissue samples were placed on the top of the Potter and, after adding 100 µL of HPLC grade water, homogenization was accomplished by moving the glass tube up and down in the ground glass pestle for about 3 minutes followed by centrifugation at 500× *g* for 2 minutes. Approximately 4 µL trypsin (40 mg/mL, in 1 mM HCl) was added to 80 µL supernatant and incubated overnight at 37 °C. The effectiveness of the high-throughput automated and manual sample preparation methods is compared in Figure 6, showing excellent similarity.

### 4.4. N-Glycan Release, Fluorophore Labeling and Sample Cleanup

A total of 80 µL ammonium acetate buffer (20 mM, pH 6.97) and 2 µL PNGase F enzyme (1.3 mg/mL) were added to the denatured samples to release the asparagine-linked glycans and incubated at 37 °C for 2 hours (Figure 1, step 3). APTS was used as a fluorophore and linked to the removed N-glycans by reductive amination. The reaction mixture contained 8 µL of THF, 7 µL of acetic acid, 3 µL of water, 2 µL of sodium-cyanoborohydride (1.0 M in THF) and 1 µL of 120 mM APTS were added to 40 µL of each sample (Figure 1, step 4). The mixture was incubated at 37 °C for 16 hours with an open cap, using our earlier published evaporative labeling protocol [42]. During the excess APTS removal step, the glycan capture magnetic bead storage liquid was replaced with HPLC water. 20 µL bead suspension was added to the dry labeled samples, followed by the addition of 185 µL of ACN. The tube containing the mixture was placed on a magnetic stand where the magnet collected the beads to one spot, allowing for the supernatant removal. A total of 20 µL of HPLC water was then added to the beads and carefully homogenized. This ACN purification step was repeated three times, and after the last ACN removal, 100 µL of water was added to release the purified APTS-labeled glycans.

### 4.5. Capillary Electrophoresis with Laser Induced Fluorescence Detection (CE-LIF)

The APTS-labeled glycans were analyzed with the CE-LIF separation method. It was accomplished in a PA 800 Plus CE Analysis System (Beckman Coulter, Brea, CA, USA) equipped with laser-induced fluorescence detection (LIF, 488 nm excitation wavelength and 520 nm emission filter) (Figure 1, step 5). A bare fused silica capillary (50 µm ID) was used with 20 cm effective length (30 cm total) and was conditioned by rinsing with HPLC-grade water for 2 min, 50% IPA (in 0.5 N HCl) for 5 min, 0.5 N NaOH for 2 min, 0.5 N HCl for 2 min, then filled with the HR-NCHO gel-buffer system. Before the sample injection a water plug was injected by applying 5 psi pressure for 5 sec, then the electrokinetic sample injection was performed with 2 kV for 2 sec in reversed polarity separation mode. The separation process was completed in HR-NCHO gel buffer at 20 °C with 30 kV applied electric potential in reversed polarity mode. All measurements were performed in triplicate, with migration time and peak area %RSD values of <1% and <5%, respectively. The generated data was collected and processed by the 32Karat software (10.1 version, Beckman Coulter).

### 4.6. Exoglycosidase-Mediated Carbohydrate Sequencing

For higher precision structural elucidation, the released and APTS-labeled glycans from the control sample were treated with an exoglycosidase enzyme array. Sequencing was carried out by the following consecutive steps using 3 × 10 µL of sample: (1) 46.2 µL HPLC water and 3.8 µL neuraminidase was added to the first aliquot; (2) 44.0 µL HPLC water, 3.8 µL neuraminidase and 2.2 µL galactosidase were added to the second aliquot, (3) 32.0 µL HPLC water, 3.8 µL neuraminidase, 2.2 µL galactosidase and 12.0 µL hexosaminidase were added to the third aliquot. The three reaction mixtures were incubated overnight at 37 °C and analyzed by CE-LIF.

To identify the peaks not shifted by the exoglycosidase array treatment in their migration time, amyloglucosidase enzyme was applied. To prepare the enzyme for digestion, the 2.0 mg amyloglucosidase was dissolved in 308 µL of ammonium acetate buffer (pH = 4.5). A total of 20 µL enzyme solution was added to 40 µL sample and incubated at 60 °C for 2 hours, then the amyloglucosidase was inactivated by incubating the reaction mixture at 90 °C for 5 minutes prior to the analysis by CE-LIF.

### 4.7. Statistical Analysis

The Kolmogorov-Smirnov test was used to analyze the distribution of the data. In case of non-normal distribution, we applied the Mann-Whitney and Wilcoxon tests to compare two groups, with *p* < 0.05 considered significant. 

## 5. Conclusions

The high-resolution glycoanalytical approach reported in this paper proved to be an efficient and sensitive workflow for glycobiomarker-based molecular diagnostics of oral malignant lesions by using the automated tissue homogenizer in combination with high-performance capillary electrophoresis analysis of the released and fluorophore-labeled N-linked glycans. Our work was based on homogeneous samples (derived from the same locus with the same histological type), but it is possible to expand to other localizations and serum or saliva for early non-invasive predictive marker discovery.

It is important to note that all results are now presented descriptively, reflecting the low number of samples, and this paper is thus considered as an exploratory characterization study. However, these preliminary results suggest that the developed workflow will allow high-throughput screening of large patient cohorts to discover and validate novel cancer-related glycobiomarkers for accurate prognosis. Future studies with individual-level data and more detailed clinical characterization are planned accordingly to further investigate these preliminary observations and clarify their biological relevance.

## Figures and Tables

**Table 1:** Identified carbohydrate structures in Figure 2. Please note that Peaks 2, 10, 13, and 19 encompassed co-migrating glycogen oligosaccharides. The structural interpretation followed the notation of Harvey et al. [37].

Peak ID	Suggested Structures (Oxford Notation Name)	Suggested Structures (Oxford Notation)
1	A2G(4)2S(3,6)2	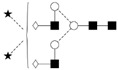
2	M3 + Glycogen	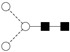
3	Glycogen	
4	F(6)A1[3]G(4)1S(6)1	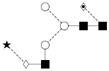
5	A2[6]G(4)1S(6)1	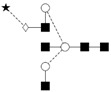
6	Glycogen	
7	Glycogen	
8	A2G(4)2S(6)1	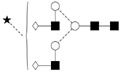
9	A2BG(4)2S(3)1	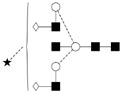
10	A2 + Glycogen	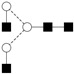
11	F(6)A2G(4)2S(3)1	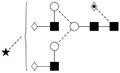
12	A2B	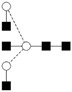
13	F(6)A2 + Glycogen	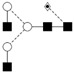
14	Glycogen	
15	A2[6]BG(4)1	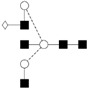
16	A2[3]BG(4)1	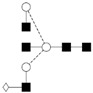
17	F(6)A2B	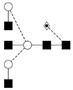
18	A2G(4)2	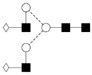
19	A2BG(4)2 + Glycogen	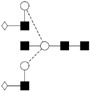
20	F(6)A2[3]BG(4)1	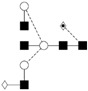
21	F(6)A2G(4)2	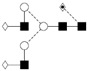
22	F(6)A2BG(4)2	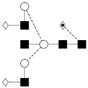
23	F(6)M3	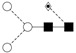

**Table 2:** Patient characteristics.

Patients	Number of Patients	Average Age (Years)	Male/Female Ratio	Localization	Metastasis	Histology	Treatment
**OSCC**	5	58.87 ± 10.37	4/1	C1 tongue	Negative	cc. planocellulare dyskeratosum G2	SOHD, hemiglossectomy, radio-chemotherapy
				C2 tongue and floor of the mouth	Negative	cc. planocellulare keratosum G1	SOHD, tumor excision, mandibular block resection, forearm flap reconstruction
				C3 radix linguae	Positive	cc. planocellulare keratosum G3	SOHD, radio-chemotherapy
				C4 tongue	Positive	cc. planocellulare keratosum G1	SOHD, mandibulotomy, radio-chemotherapy
				C5 tongue	Negative	cc. planocellulare dyskeratosum G3	SOHD, hemiglossectomy
**Control**	7	50.86 ± 3.2	4/3				

## Data Availability

The data presented in this study are available upon request from the corresponding author.

## Supplementary Materials

The following supporting information can be downloaded at: https://www.mdpi.com/article/10.3390/ijms27020740/s1.

## Abbreviations

The following abbreviations are used in this manuscript:

APTS	1-Aminopyrene-3,6,8-trisulfonate
ACN	Acetonitrile
BAEE	Nα-benzoyl-L-arginine ethyl ester
CE-LIF	Capillary electrophoresis with laser induced fluorescence detection
FUT6	Fucosyltransferase enzyme
HILIC	Hydrophilic interaction liquid chromatography
HPLC	High-performance liquid chromatography
HPV	Human papilloma virus
HR-NCHO	High-resolution N-linked carbohydrate separation buffer
ITN	Intratracheal narcosis
MS	Mass spectrometry
OP-SCC	Oropharyngeal squamous cell carcinoma
OSCC	Oral squamous cell carcinoma
OSCC-C	Oral squamous cell carcinoma-control
OSCC-T	Oral squamous cell carcinoma-tumor
PGC-LC	Porous graphitized carbon liquid chromatography
PNGase F	Peptide N-Glycosidase F enzime
SF/NF	Sialoform to neutral carbohydrate ratio
SOHD	Supraomohyoid neck dissection
TG	Total N-glycan
THF	Tetrahydrofuran
