# Supplementary material for: Analysis of Soft Tissue N-Glycome Profiles in Oral Squamous Cell Carcinoma, a Pilot Study
Source: Int J Mol Sci. 2026 Jan 11;27(2):740. doi: 10.3390/ijms27020740 (PMC12841353; doi:10.3390/ijms27020740)
Supplement: Supplementary file 1 [file ijms-27-00740-s001.zip › ijms-4046687-supplementary.pdf]

# Analysis of soft tissue N-glycome profiles in Oral Squamous Cell Carcinoma, a pilot study

**Eniko Gebri[1], Kinga Hogyor[2], Adrienne Szabo[3] and Andras Guttman[2,4*]**

[1] Department of Oral Medicine, Faculty of Dentistry, University of Debrecen, Debrecen, Hungary; gebri.eniko@dental.unideb.hu

[2] Translational Glycomics Group, Research Institute of Biomolecular and Chemical Engineering, University of Pannonia, Veszprem, Hungary; hogyorkinga@gmail.com

[3] Department of Oral and Maxillofacial Surgery, Faculty of Dentistry, University of Debrecen, Debrecen, Hungary; szabo.adrienn@dental.unideb.hu

[4] Horváth Csaba Memorial Laboratory of Bioseparation Sciences, Research Center for Molecular Medicine, Faculty of Medicine, University of Debrecen, Hungary; guttmanandras@med.unideb.hu

*Correspondence: guttmanandras@med.unideb.hu; orcid.org/0000-0002-7838-082X

**Supplementary Table S1.** The identified soft tissue carbohydrate structures (N-glycans and glycogens) and the corresponding relative peak area % values in the healthy volunteers (C) as well as the malignant (OSCC-T) and adjacent (OSCC-C) gingival side of patients with oral squamous cell carcinoma.

| *Peak #* | **Structure** | **Peak area%** | | | | | | | | | | | | | | | |
|---|---|---|---|---|---|---|---|---|---|---|---|---|---|---|---|---|---|
| | | **C1** | **C2** | **C3** | **C4** | **C5** | **C6** | **C7** | **OSCC-C1** | **OSCC-C2** | **OSCC-C3** | **OSCC-C4** | **OSCC-C5** | **OSCC-T1** | **OSCC-T2** | **OSCC-T4** | **OSCC-T5** |
| 1 | A2G(4)2S(3,6)2 | 6.66 | 7.23 | 5.38 | 2.80 | 2.24 | 4.42 | 4.84 | 4.87 | 4.25 | 7.26 | 12.91 | 0.80 | 4.74 | 11.72 | 11.53 | 2.38 |
| 2 | M3 (+Glycogen) | 2.70 | 2.43 | 3.47 | 2.94 | 2.06 | 3.05 | 3.44 | 2.58 | 4.55 | 5.52 | 5.62 | 2.38 | 3.08 | 4.02 | 6.49 | 4.00 |
| 3 | Glycogen | 4.11 | 2.38 | 7.61 | 3.91 | 4.44 | 4.30 | 5.04 | 3.42 | 8.83 | 11.69 | 6.46 | 7.87 | 6.84 | 8.99 | 6.90 | 17.59 |
| 4 | F(6)A1[3]G(4)1S(6)1 | 0.63 | 0.21 | 0.46 | 0.73 | 0.05 | 1.30 | 0.78 | 1.89 | 0.74 | 1.41 | 0.30 | 0.63 | 2.71 | 4.95 | 1.84 | 12.26 |
| 5 | A2[6]BG(4)1S(6)1 | 1.21 | 1.02 | 0.40 | 0.97 | 1.92 | 1.10 | 0.26 | 1.35 | 0.42 | 1.69 | 3.09 | 0.19 | 0.74 | 2.21 | 0.44 | 0.84 |
| 6 | Glycogen | 5.61 | 8.16 | 15.33 | 12.38 | 13.42 | 6.44 | 14.13 | 4.80 | 22.33 | 16.68 | 14.13 | 24.94 | 8.24 | 7.81 | 12.81 | 16.46 |
| 7 | Glycogen | 2.89 | 2.11 | 2.79 | 2.54 | 1.83 | 3.12 | 3.22 | 2.17 | 4.16 | 3.63 | 3.35 | 4.97 | 2.48 | 1.87 | 5.01 | 4.04 |
| 8 | A2G(4)2S(6)1 | 13.99 | 17.64 | 10.90 | 7.91 | 6.09 | 9.41 | 10.75 | 10.36 | 3.58 | 4.96 | 8.64 | 2.08 | 9.77 | 7.91 | 8.58 | 4.94 |
| 9 | A2BG(4)2S(3)1 | 1.06 | 1.20 | 0.51 | 0.47 | 0.60 | 0.74 | 0.42 | 0.89 | 0.08 | 0.50 | 0.87 | 0.16 | 0.41 | 0.43 | 1.09 | 0.22 |
| 10 | A2 (+Glycogen) | 7.62 | 8.84 | 13.34 | 14.86 | 19.42 | 8.40 | 12.69 | 7.52 | 19.66 | 12.27 | 15.59 | 28.47 | 9.85 | 8.10 | 11.16 | 7.63 |
| 11 | F(6)A2G(4)2S(3)1 | 6.33 | 6.70 | 4.81 | 6.30 | 11.04 | 6.02 | 6.73 | 5.65 | 5.19 | 3.08 | 6.12 | 3.54 | 5.05 | 10.29 | 4.44 | 5.90 |
| 12 | A2B | 0.62 | 0.30 | 0.33 | 0.32 | 0.44 | 0.99 | 0.58 | 0.63 | 0.45 | 0.98 | 0.37 | 0.76 | 1.50 | 1.79 | 1.11 | 2.43 |
| 13 | F(6)A2 (+Glycogen) | 13.00 | 7.92 | 8.73 | 11.93 | 8.27 | 14.56 | 9.48 | 14.54 | 9.50 | 8.62 | 9.80 | 9.90 | 10.48 | 8.03 | 9.35 | 3.97 |
| 14 | Glycogen | 1.29 | 1.46 | 1.40 | 1.50 | 1.20 | 1.22 | 1.56 | 1.24 | 1.37 | 1.08 | 1.35 | 2.23 | 1.83 | 0.92 | 2.23 | 2.04 |
| 15 | A2[6]BG(4)1 | 2.48 | 1.54 | 1.71 | 1.77 | 1.03 | 3.07 | 2.24 | 2.96 | 0.89 | 2.69 | 0.90 | 0.25 | 2.66 | 1.28 | 1.50 | 1.67 |
| 16 | A2[3]BG(4)1 | 0.83 | 0.49 | 1.12 | 1.19 | 1.52 | 1.55 | 1.76 | 0.52 | 1.75 | 1.86 | 0.66 | 0.18 | 0.89 | 2.28 | 1.43 | 1.13 |
| 17 | F(6)A2[6]G(4)1 | 4.78 | 3.13 | 2.90 | 3.70 | 1.81 | 5.76 | 3.15 | 7.67 | 2.15 | 3.31 | 1.81 | 2.22 | 5.57 | 3.20 | 2.77 | 1.94 |
| 18 | A2G(4)2 | 9.55 | 12.92 | 7.76 | 7.07 | 5.23 | 9.52 | 7.38 | 10.90 | 2.38 | 2.48 | 2.74 | 1.06 | 8.37 | 2.89 | 3.33 | 1.21 |
| 19 | A2BG(4)2 | 0.97 | 1.15 | 1.57 | 1.79 | 1.74 | 1.27 | 1.52 | 0.85 | 2.11 | 1.84 | 0.99 | 1.41 | 1.73 | 0.79 | 1.58 | 2.57 |
| 20 | F(6)A2[3]BG(4)1 | 4.68 | 2.11 | 2.52 | 2.98 | 0.81 | 5.05 | 3.97 | 3.56 | 1.01 | 4.38 | 0.30 | 2.43 | 4.36 | 5.35 | 2.24 | 2.49 |
| 21 | F(6)A2G(4)2 | 7.74 | 9.83 | 5.40 | 10.18 | 12.03 | 7.21 | 4.82 | 10.22 | 3.02 | 3.14 | 3.25 | 1.17 | 7.05 | 4.88 | 3.00 | 2.50 |
| 22 | F(6)A2BG(4)2 | 1.27 | 1.26 | 1.58 | 1.75 | 2.81 | 1.48 | 1.26 | 1.41 | 1.57 | 0.95 | 0.77 | 2.34 | 1.64 | 0.29 | 1.18 | 1.79 |

**Supplementary Table S2.** Comparison of the identified soft tissue carbohydrate structures (N-glycans and glycogens) in the examined groups (healthy volunteers (C), malignant (OSCC-T) and adjacent (OSCC-C) gingival side of patients with oral squamous cell carcinoma) evaluated with the Mann-Whitney (C vs OSCC-C and C vs OSCC-T) and Wilcoxon test (OSCC-C vs OSCC-T). The red text depicts the structures where $p \leq 0.05$.

| *peak #* | **Glycan Structure** | **C vs OSCC-C** | **C vs OSCC-T** | **OSCC-C vs OSCC-T** |
|---|---|---|---|---|
| 1 | A2G(4)2S(3,6)2 | 0.7551 | 0.5273 | 0.0625 |
| 2 | M3 (+Glycogen) | 0.3434 | *0.0242* | 0.0625 |
| 3 | Glycogen | 0.1061 | *0.0242* | 0.0625 |
| 4 | F(6)A1[3]G(4)1S(6)1 | 0.2677 | *0.0061* | *0.0258* |
| 5 | A2[6]BG(4)1S(6)1 | 0.6389 | 0.9273 | 0.125 |
| 6 | Glycogen | 0.202 | 0.7879 | 0.125 |
| 7 | Glycogen | *0.048* | 0.6485 | 0.125 |
| 8 | A2G(4)2S(6)1 | *0.048* | 0.2303 | 0.0625 |
| 9 | A2BG(4)2S(3)1 | 0.4318 | 0.2303 | 0.0625 |
| 10 | A2 (+Glycogen) | 0.4318 | 0.3152 | 0.125 |
| 11 | F(6)A2G(4)2S(3)1 | *0.0303* | 0.3152 | 0.0625 |
| 12 | A2B | 0.2677 | *0.0061* | 0.0625 |
| 13 | F(6)A2 (+Glycogen) | *0.0025* | *0.0061* | 0.0625 |
| 14 | Glycogen | 0.4318 | 0.5273 | 0.125 |
| 15 | A2[6]BG(4)1 | 0.7551 | 0.7879 | 0.0625 |
| 16 | A2[3]BG(4)1 | 0.5303 | 0.7879 | 0.125 |
| 17 | F(6)A2[6]G(4)1 | 0.0732 | 0.0727 | 0.125 |
| 18 | A2G(4)2 | >0,9999 | 0.6485 | 0.0625 |
| 19 | A2BG(4)2 + Glycogen | 0.4318 | 0.7879 | 0.125 |
| 20 | F(6)A2[3]BG(4)1 | 0.4318 | 0.7879 | 0.0625 |
| 21 | F(6)A2G(4)2 | 0.0732 | *0.0424* | 0.0563 |
| 22 | F(6)A2BG(4)2 | 0.5303 | 0.6485 | 0.125 |

**Supplementary Table S3.** The relative peak area % of the identified sialylated, fucosylated, neutral N-glycan structures and the totals in the three examined groups (healthy volunteers (C), malignant (OSCC-T) and adjacent (OSCC-C) gingival side of patients with oral squamous cell carcinoma).

| **Sialylated structures** | **C** | **OSCC-C** | **OSCC-T** |
|---|---|---|---|
| A2G(4)2S(3,6)2 | 4.79 | 6.02 | 7.59 |
| F(6)A1[3]G(4)1S(6)1 | 0.59 | 0.99 | 5.44 |
| A2[6]BG(4)1S(6)1 | 0.98 | 1.35 | 1.06 |
| A2G(4)2S(6)1 | 10.96 | 5.92 | 7.80 |
| A2BG(4)2S(3)1 | 0.72 | 0.50 | 0.54 |
| F(6)A2G(4)2S(3)1 | 6.85 | 4.72 | 6.42 |
| ***MEAN*** | **4.15** | **3.25** | **4.81** |
| ***SD*** | **4.21** | **2.58** | **3.23** |
| | | | |
| **Fucosylated structures** | **C** | **OSCC-C** | **OSCC-T** |
| F(6)A1[3]G(4)1S(6)1 | 0.59 | 0.99 | 5.44 |
| F(6)A2G(4)2S(3)1 | 6.85 | 4.72 | 6.42 |
| F(6)A2 (+Glycogen) | 3.17 | 3.14 | 2.39 |
| F(6)A2[6]G(4)1 | 3.60 | 4.50 | 3.37 |
| F(6)A2[3]BG(4)1 | 3.16 | 2.34 | 3.61 |
| F(6)A2G(4)2 | 8.17 | 4.16 | 4.36 |
| F(6)A2BG(4)2 | 1.63 | 1.41 | 1.09 |
| ***MEAN*** | **3.88** | **3.04** | **3.81** |
| ***SD*** | **2.71** | **1.50** | **1.80** |
| | | | |
| **Neutral structures** | **C** | **OSCC-C** | **OSCC-T** |
| A2 (+Glycogen) | 12.17 | 16.70 | 9.18 |
| A2B | 0.51 | 0.64 | 1.71 |
| A2[6]BG(4)1 | 1.98 | 1.54 | 1.78 |
| A2[3]BG(4)1 | 1.21 | 2.19 | 1.43 |
| F(6)A2[6]G(4)1 | 3.60 | 4.50 | 3.37 |

| | | | |
|---|---|---|---|
| A2G(4)2 | 8.49 | 3.91 | 3.95 |
| A2BG(4)2 + (Glycogen) | 4.66 | 2.56 | 2.63 |
| F(6)A2[3]BG(4)1 | 3.16 | 2.34 | 3.61 |
| F(6)A2G(4)2 | 8.17 | 4.16 | 4.36 |
| F(6)A2BG(4)2 | 1.63 | 1.41 | 1.09 |
| ***MEAN*** | **4.56** | **4.15** | **3.39** |
| ***SD*** | **3,83** | **4.64** | **2.36** |
| | | | |
| **Total** | **C** | **OSCC-C** | **OSCC-T** |
| A2G(4)2S(3,6)2 | 4.79 | 6.02 | 7.59 |
| M3 (+Glycogen) | 4.79 | 6.02 | 7.59 |
| F(6)A1[3]G(4)1S(6)1 | 0.59 | 0.99 | 5.44 |
| A2[6]BG(4)1S(6)1 | 0.98 | 1.35 | 1.06 |
| A2G(4)2S(6)1 | 10.96 | 5.92 | 7.80 |
| A2BG(4)2S(3)1 | 0.72 | 0.50 | 0.54 |
| A2 (+Glycogen) | 12.17 | 16.70 | 9.18 |
| F(6)A2G(4)2S(3)1 | 6.85 | 4.72 | 6.42 |
| A2B | 0.51 | 0.64 | 1.71 |
| F(6)A2 (+Glycogen) | 3.17 | 3.14 | 2.39 |
| A2[6]BG(4)1 | 1.98 | 1.54 | 1.78 |
| A2[3]BG(4)1 | 1.21 | 2.19 | 1.43 |
| F(6)A2[6]G(4)1 | 3.60 | 4.50 | 3.37 |
| A2G(4)2 | 8.49 | 3.91 | 3.95 |
| A2BG(4)2 | 3.69 | 3.06 | 2.63 |
| F(6)A2[3]BG(4)1 | 3.16 | 2.34 | 3.61 |
| F(6)A2G(4)2 | 8.17 | 4.16 | 4.36 |
| F(6)A2BG(4)2 | 1.63 | 1.41 | 1.09 |
| ***MEAN*** | **4.30** | **3.84** | **4.00** |
| ***SD*** | **3.62** | **3.71** | **2.72** |

**Supplementary Table S4.** The sialoform to neutral (SF/NF) and the core-fucosylated to total carbohydrate ratio of the identified soft tissue N-glycans in the examined groups (healthy volunteers (C), malignant (OSCC-T) and adjacent (OSCC-C) gingival side of patients with oral squamous cell carcinoma).

| | **C (mean)** | **SD** | **OSCC-C (mean)** | **SD** | **OSCC-T (mean)** | **SD** | ***p* value** | |
|---|---|---|---|---|---|---|---|---|
| **SF** | 4.15 | 4.21 | 3.25 | 2.58 | 4.81 | 3.23 | **C vs OSCC-C** | 0.86 |
| **NF** | 4.56 | 3.83 | 3.99 | 4.64 | 3.31 | 2.36 | **C vs OSCC-T** | 0.46 |
| **ratio%** | **0.91** | **1.09** | **0.81** | **0.56** | **1.45** | **1.37** | **OSCC-C vs OSCC-T** | 0.36 |
| | | | | | | | | |
| | **C (mean)** | **SD** | **OSCC-C (mean)** | **SD** | **OSCC-T (mean)** | **SD** | ***p* value** | |
| **CF** | 3.88 | 2.71 | 3.04 | 1.50 | 3.81 | 1.80 | **C vs OSCC-C** | 0.77 |
| **TG** | 4.30 | 3.62 | 3.84 | 3.71 | 4.00 | 2.72 | **C vs OSCC-T** | 0.91 |
| **ratio%** | **0.9** | **0.74** | **0.79** | **0.4** | **0.95** | **0.66** | **OSCC-C vs OSCC-T** | 0.66 |
